# A Generative Adversarial Network (GAN) Technique for Internet of Medical Things Data

**DOI:** 10.3390/s21113726

**Published:** 2021-05-27

**Authors:** Ivan Vaccari, Vanessa Orani, Alessia Paglialonga, Enrico Cambiaso, Maurizio Mongelli

**Affiliations:** 1 Consiglio Nazionale delle Ricerche (CNR), Institute of Electronics, Information Engineering and Telecommunications (IEIIT), 16149 Genoa, Italy; vanessa.orani@ieiit.cnr.it (V.O.); enrico.cambiaso@ieiit.cnr.it (E.C.); maurizio.mongelli@ieiit.cnr.it (M.M.); 2 Consiglio Nazionale delle Ricerche (CNR), Institute of Electronics, Information Engineering and Telecommunications (IEIIT), 20133 Milan, Italy; alessia.paglialonga@ieiit.cnr.it

**Keywords:** Internet of Medical Things (IoMT), generative adversarial networks (GANs), healthcare, machine learning, intelligible analytics, statistical validation, remote monitoring

## Abstract

The application of machine learning and artificial intelligence techniques in the medical world is growing, with a range of purposes: from the identification and prediction of possible diseases to patient monitoring and clinical decision support systems. Furthermore, the widespread use of remote monitoring medical devices, under the umbrella of the “Internet of Medical Things” (IoMT), has simplified the retrieval of patient information as they allow continuous monitoring and direct access to data by healthcare providers. However, due to possible issues in real-world settings, such as loss of connectivity, irregular use, misuse, or poor adherence to a monitoring program, the data collected might not be sufficient to implement accurate algorithms. For this reason, data augmentation techniques can be used to create synthetic datasets sufficiently large to train machine learning models. In this work, we apply the concept of generative adversarial networks (GANs) to perform a data augmentation from patient data obtained through IoMT sensors for Chronic Obstructive Pulmonary Disease (COPD) monitoring. We also apply an explainable AI algorithm to demonstrate the accuracy of the synthetic data by comparing it to the real data recorded by the sensors. The results obtained demonstrate how synthetic datasets created through a well-structured GAN are comparable with a real dataset, as validated by a novel approach based on machine learning.

## 1. Introduction

Chronic Obstructive Pulmonary Disease (COPD) is a critical pulmonary disease affecting about 5–10% of the adult population [1] and is associated with significant healthcare and socioeconomic burden; thus, it is important to carefully prevent and monitor such disease. In recent years, an important topic in healthcare is related to remote monitoring of patients. Remote monitoring of diseases and treatments is based on the collection of vital parameters (such as heart rate, blood oxygenation, sleep and activity of the patient) necessary to evaluate possible immediate and timely medical interventions to avoid worsening of the disease and related clinically relevant symptoms and, in general terms, to improve the quality of life of patients, their families and the population [2,3,4].

Within this umbrella, we propose a new implementation for remote monitoring through Pneulytics, a platform to monitor and manage patients with COPD [5]. Pneulytics can be used by health systems, clinical centers, pulmonology wards and clinics. Platforms that include patient devices to retrieve patient routine data are not yet widely adopted, but billions of Internet of Medical Things (IoMT) devices are expected to be used in this rapidly growing application field [6]. The platform is able to collect clinical data from IoMT devices to analyze such data using artificial intelligence (AI) algorithms.

Moreover, data must be well structured and in large quantities to develop accurate and precise algorithms capable of identifying the disease timely and precisely to prevent possible serious consequences. However, in real-world settings, the amount of data collected through monitoring devices may be insufficient to train predictive algorithms, for example, due to irregular use of devices, misuse, or lack of compliance and adherence to the monitoring program [7]. Data augmentation can potentially help mitigate these problems. Data augmentation techniques are often used to generate new data by transforming existing datasets or by generating new synthetic data, always starting from real and existing data [8]. In healthcare, data augmentation has been applied, for example, to signals and images to improve disease detection and prediction [9,10,11,12]. In order to achieve this purpose, in recent years, a new concept called Generative Adversarial Networks (GAN) has emerged that offers an innovative method for data augmentation [13]. In the field of machine learning, two neural networks, called discriminator and generator, are trained to generate new data having the same distribution as the initial dataset [14]. GAN shows promising results in image generation tasks, and as such, it has been applied in image-related problems such as texture synthesis, domain translation, and image completion [15,16,17,18]. For example, with GAN, it is possible to obtain a neural network capable of generating hyper-realistic human faces, as demonstrated in different research works [19,20,21].

In the healthcare world, GAN techniques are mainly used for the generation of new synthetic health data, mainly used in AI projects and activities [22]. The main goal of this application is the generation of new data for machine learning (ML) and AI algorithms to make up for the possibility of a lack of data. The use of GAN would lead to a large production of data in compliance with the criticalities of the case. Furthermore, these data can be shared between various entities as being synthetic, and they do not contain personal data, thus privacy issues could vanish.

Given the importance of a sufficiently large dataset in the healthcare context to implement refined machine learning algorithms capable of identifying diseases according to different vital parameters, we decided to use a GAN algorithm to perform data augmentation on a small initial dataset. The purpose of this process is to simulate additional individuals monitored in their different vital parameters to verify how the machine learning algorithm responds and improves itself with a greater amount of data (obviously, the final purpose is to refine the symptoms/pathology identification mechanism).

The remainder of the paper is structured as follows: Section 2 reports an outline of the research gap, giving a clear context to the objectives of the work. Section 3 deeply describes the proposed Pneulytics framework, the data collection approach and IoMT devices. Section 4 details the artificial intelligence algorithm, while Section 5 introduces an application of data augmentation and discusses the possible next steps in this topic. Finally, Section 8 concludes the paper.

## 2. Research Gap

Today, more than ever, due to population aging, the increasing burden of chronic conditions and multimorbidity in older adults, and the COVID-19 pandemic, the healthcare systems need to be dramatically innovated, especially by the introduction of technologies that may enable organized and decentralized management of patients, particularly of those who need continuous follow up due to their chronic conditions (e.g., [23,24]). Recently, we have witnessed a rapid evolution in the fields of the Internet of Medical Things (IoMT), data processing and fusion, and telemedicine [25]. The field of IoMT has opened new opportunities for patient monitoring and remote service provision, particularly in older adults with chronic diseases [26,27]. However, the rise of the IoMT has also brought new challenges in terms of, for example, privacy, security, and computing capabilities [28]. Nonetheless, the current availability of multi-sensors systems natively integrated with AI is scarce. Currently available telemedicine systems are capable of sensing and communication through a data hub, but they typically have limited processing capabilities (e.g., detection of parameters exceeding desired ranges to deliver alerts) [29]. These simplified, one-size-fits-all alert systems may not be able to effectively support physicians in monitoring their patients as specific patients may have specific ‘desired’ ranges for their biomarkers, and physicians can obtain a limited insight into the patient status. There is an urgent need for novel, intelligent systems able to monitor patients in an individualized way. The integration of AI algorithms, specifically explainable AI (xAI) methods that are able to generate predictive models of the patient’s health, can introduce a revolution in this field [30,31]. The generation of models in the form of intelligible rules (if-then-else) can help physicians understand the factors leading to better/worse outcomes in their patients and, also, can help identify countermeasures to limit the risk of deterioration by modifying specific biomarkers, as identified by the model rules, to lead them within the desired limits. This type of approach not only enables a deeper understanding of the factors that determine the evolution of the patient status but it also enables the identification of specific countermeasures and therapeutic approaches on an individual basis.

## 3. Pneulytics Framework

The Pneulytics framework is aimed at monitoring patients affected by COPD by combining innovative technologies and artificial intelligence algorithms. COPD is a common, widespread, preventable and treatable disease associated with persistent respiratory symptoms (such as dyspnoea, cough, expectoration) and airway obstructions due to induced lung damage, such as from cigarette smoke and environmental pollutants. COPD patients are generally treated with topically administered medications, such as transdermal patches or small adhesive strips, to avoid worsening and relieve symptoms and increase lung function. Unfortunately, nowadays, it is difficult to actually assess how consistent patient outcomes in clinical research and in real life are, mainly due to poor adherence to treatments and misuse of drug inhalers [32]. Failure to use inhalers is associated with an increase in possible relapses and worsening of COPD, and the combination of poor adherence and misuse of inhalers can increase the risk of death [32,33].

In recent years, technological improvements have been introduced that allow remote patient monitoring and enable direct interaction between patients and healthcare professionals [34,35]. For example, there are smart inhalers that can record and digitize key aspects of patient monitoring, such as medication intake and parameters related to proper use of the inhaler (e.g., peak inspiratory flow, duration of inspiratory phase, orientation of the inhaler). Through these smart inhalers, doctors are able to acquire information on treatment adherence and correct/improper use of the inhaler and then monitor the patient in detail. The Pneulytics framework integrates the analysis of smart inhaler data with the analysis of patient monitoring data from IoMT and clinical data from follow-up visits.

**Figure 1:**
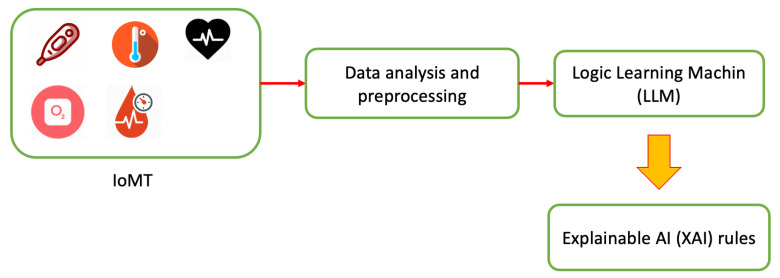
Pneulytics framework architecture.

Despite technological advances, it is not yet possible to carry out an accurate analysis of the health of patients by carrying out remote monitoring. To achieve a fair level of monitoring of patients’ vital parameters, the Pneulytics framework aims to combine IoMT devices with innovative artificial intelligence algorithms with the aim of preventing and treating diseases, in this specific case COPD, through remote monitoring, performed directly by doctors and health entities. The Pneulytics prototype is composed of some hardware components used for the collection of patient parameters, obtained through IoMT devices, and a software part for the accurate processing of the data recovered from the sensors through machine learning algorithms, specifically explainable AI [5], combined with historical data. In the previous work, data were focused only on a smart inhaler, while in this work, we elaborated data retrieved by other IoMT as described in Section 3.1. The proposed technological platform is shown in Figure 1.

In detail, patients wear sensors for monitoring parameters relating to the disease under analysis during their day. All sensors are currently connected to a smartphone, which periodically sends data to the cloud platform where the data are stored. In combination with patient sensors, environmental sensors will be introduced to assess how the living environment can affect the disease. Subsequently, after retrieving patient data, ad hoc AI algorithms are performed to develop predictive models that can be used to identify, define and prevent possible diseases. Furthermore, doctors, having direct access to the data and results of AI algorithms, can prepare medical plans and any interventions on therapies.

### 3.1. Adopted IoT Devices

The aim of our research is to investigate the adoption of smart building and health devices for monitoring purposes, especially regarding quality of life and wellbeing measurements. For instance, Indoor Environmental Quality (IEQ) is represented by a collection and combination of different measurements to identify the quality of an indoor environment. With this purpose, we set up an intelligent monitoring system able to observe, capture and process both environmental and body measurements. Thanks to the Internet of Things sensors, it is possible to retrieve and exchange such data easily, due to the interconnection between the sensors and a common data storage platform.

Considering the set of IoT sensors adopted for the platform, we have focused on the possibility of accessing raw data captured by the sensors. Particularly, we focused on both wearable devices, connected and actively managed by the patients, and environmental devices, physically installed at the patients’ home, which will be part of a future version of the platform.

Regarding wearable IoMT devices, for the first proof-of-concept study, we adopted the H&S cloud platform HealthPlatform v3 (CompuGroupMedical), which is able to access aggregated monitoring data and provide authorized access to the end user. The H&S proprietary platform is certified medical device CE IIA and equipped with a data center certified ISO 27001 and ISO 13485. Data management activities are compliant with GDPR and CE Medical Device 5/2020.

Among the available devices, our proof-of-concept platform includes:

- A dedicated smartphone with the proprietary app (Mhealth, certified IIA class) running on it,
- An electrocardiogram (ECG) patch also providing day/night movement monitoring,
- A pulse meter providing oximetry monitoring,
- A weight scale,
- A sphygmomanometer for blood pressure monitoring,
- A spirometer for peak flow and FEV1 parameters.

Communication between sensors and mobile devices is based on Bluetooth Low Energy, a protocol used in the IoT context [36]. In particular, the patient wears the medical sensor and starts monitoring the associated health parameter. The sensor autonomously connects to the mobile device and sends data to it. Finally, the mobile device displays the measurement and uploads it directly to the cloud for data storage. The list of devices reported is the complete one of the Pneulytics architecture, but in this work, we have only used some of the devices mentioned; in particular, the pulse meter, the weight scale, the sphygmomanometer, the spirometer and the smartphone. In future versions, the smartphone will also be used to set up validated questionnaires for the patients, such as the COPD assessment test.

Regarding environmental monitoring, our setup includes, for each environment (home, office, etc.),

- A central node receiving data from the other nodes,
- Physical sensors distributed in the environment able to retrieve different measures,
- A set of modules demanded to provide connectivity to analog equipment like windows or radiators,
- An outdoor weather station.

Particularly, while the central node is represented by a Raspberry Pi 4 Model B, the following sensor types and models are considered:

- Temperature and humidity (Sparkfun, SI7021);
- Atmospheric pressure (AZ Delivery, BMP180);
- Air speed (Modern Device, Wind Sensor Rev. C);
- CO measurement (Sparkfun, MQ7);
- CO${}_{2}$ measurement (Sparkfun, CSS811);
- Formaldehyde concentration (Seeedstudio, Grove HCHO);
- Concentration of fine dust (Honeywell, HPMA115S0-XXX);
- Redundancy (Bosch, BME680);
- Weather station (PCE Italia, PCE FWS 20).

Finally, regarding analog equipment monitoring, we used AZDelivery, ESP8266 plus ESP-01 and DHT22 plus AM2302. The communication between sensors and the central node is based on Message Queuing Telemetry Transport (MQTT), an ad hoc IoT communication protocols. Sensors distributed in the environment retrieve information every 5 min and send the data to the central node. The central node encapsulates the data into a single packet and sends the entire packet to a web server every 15 min. The data retrieved by the ambient sensors are not considered in this work since we are implementing the system, but they will be the scope of future works.

Thanks to the distributed sensors, we are able to observe and process multiple metrics, both referring to the conditions of the patients and the underlying environment. In addition, data aggregation and processing through AI methodologies is able not only to combine information from different components for a common analysis, but also to identify potential relationships among the data.

## 4. The Artificial Intelligence Approach

The information acquired on the IoMT platform is exploited to infer knowledge extraction of the quality of the medical treatment through AI. This implies a machine learning formulation of the problem.

A prediction function f((·),·) is defined in order to model the mapping between collections of sensor measurements and their impact on the evolution of the disease over time. A canonical supervised learning problem is posed to this aim, whose target is a medical key performance indicator, coherent with the disease of interest.

The techniques refer to *eXplainable Artificial Intelligence* (XAI) (or *intelligible analytics*), which means understandable machine learning. It helps human experts of the field enter the logic of the machine learning process and elaborate the knowledge extraction even more (cognitive machine learning). An example of this is argument of the following performance evaluation, in which rules are compared and understood under different conditions of the reference dataset (baseline versus data augmentation).

Technically speaking, a supervised classification problem consists of finding the best boundary function Ψ(x) separating points in classes (good and bad treatment here). Under the XAI paradigm, the Ψ(x) model is described by a set of *m* intelligible rules $r_{k},k=1,\ldots ,m$, of the type **if** (*premise*) **then** (*consequence*), where (*premise*) is a logical product (AND, ∧) of $d_{k}$ conditions $c_{l_{k}},\;\mathrm{with}\;l_{k}=1_{k},\ldots ,d_{k}$, and (*consequence*) provides a class assignment for the output, i.e., $\hat{y}=g\left(x\right)$.

The decision making process of Decision Tree (**DT**) consists of splitting the dataset available at the node into two subsets (two branch nodes) according to a specific criterion. Statistical metrics, such as information gain, define the criteria and infer the quality of a split at a node. The logic behind the decision tree can be easily understood because it shows a tree-like structure that, in turn, can be easily converted into rules (chapter 9 of [37]).

The Logic Learning Machine (**LLM**) [31] deals with clustering data samples after mapping them into a boolean space (latticization) from which the derivation of rules is readly available. The discretization of the data space before latticization is essential to reduce quantity of information, still preserving the quality of it [38], thus opening the door to big data support.

Differently from DT [37], in which some polarization on most meaningful features and low robustness to unbalanced datasets may arise, the LLM clusterization process helps to look at the underlying phenomenon under different angles, still joining all the variables involved. From the preliminary results, reported in [30], the LLM revealed very good results compared with those of the most known learning techniques. Moreover, the computational complexity of the method is kept low through the adoption of proper greedy procedures. Therefore, the LLM model may also be adopted in the analysis of large datasets (i.e., having many inputs and/or examples). Notice that the LLM approach presents further interesting features such as the possibility of dealing with categorical inputs and the determination of the relevance of each variable. This last property allows the identification and elimination of redundant attributes.

### 4.1. LLM Computational Cost

The LLM firstly converts original data into binary strings (Boolean space) and then maps groups of strings into rules. The rule extraction process is based on clustering of strings in the new space. The resulting machine learning model consists of the overall set of extracted rules. The granularity of the clusters is proportional to the precision (and potential overfitting) of the model and inversely proportional to the generalization to new data. Therefore, a trade-off is typically considered between precision and generalization by accepting clusters large enough to contain a small classification error. That means a cluster contains points of a given class in prevalence, but it also contains a small proportion of points of the other classes. Overlapping classes in large clusters lead to less precision but higher generalization and more synthesis of the model.

Since every cluster is later converted into a rule, the most significant rules (i.e., rules with higher covering of data) are derived from large clusters and vice-versa. Small clusters are of interest however, as they may provide rules representing anomalies or singularities in the data. The LLM setting, therefore, follows those principles by tuning the number of extracted rules as a trade-off between synthesis and generalization (fewer rules with high covering) and precision (more rules with low covering).

The supplementary material of [39] may help with the understanding of this, as it shows an example of binarization, clustering and rule extraction. The setting of the overall process has an impact in computational terms as well. More precision requires a higher computational cost. Mathematically speaking, Section VI.A of [31] deals with closed-form expressions of the computational cost that approximately follows O(m^2^ n^2^), where n is the length of the binary strings and m is the size of the training set. In practice, manual inspection is typically applied in order to stop the computation when a plateau becomes evident in terms of precision, number and covering of the rules.

### 4.2. Data Collection and Validation

This section reports the results obtained by monitoring patients through the devices outlined in Section 3.1. A single COPD subject is considered, and we provide analysis in terms of data augmentation. Moreover, we show how GAN could be used to perform a data augmentation approach. Such indicators are still considered statistically valid and suitable for further interpretation by the medical staff. We firstly start with data collection without adversarial machine learning and show the baseline rules available from LLM inference. Once more, it is worth noting that XAI offers support to the understanding of model evolution under different conditions (baseline vs. augmentation).

The following data are structured in a database for further LLM inference (the Rulex platform has been used: http://www.rulex.ai, accessed on 26 May 2021). Data are collected every day for three consecutive months: oxygen, body temperature, heart rate, heart rate master, weight, Body Mass Index, FEV1, PEF, MAP, diastolic blood pressure, systolic blood pressure. FEV1 is the forced expiratory volume in 1 s, i.e., the volume of air (in liters) exhaled in the first second during forced exhalation after maximal inspiration. PEF is the Peak Expiratory Flow, i.e., the maximum flow (or velocity) that can be achieved when performing a forced exhalation that is initiated after a full inspiration, measured in liters per minute or liters per second. As an alternative to systolic blood pressure, MAP deals with the average artery pressure on a cardiac cycle. Two sources of information are available for the heart rate as well: from the oximeter and the sphygmomanometer; the feature corresponding to the latter device is denoted by the suffix ’master’ (i.e., heart rate master).

The classification problem deals with PEF under and over a threshold of 400 L per minute.

As each rule defines a specific stratification of data, the potential statistical significance should be assessed. The validation procedure follows [5] by exploiting the Fisher’s Exact Test (FET), which is suitable for a small amount of data.

### 4.3. Baseline

Real data are collected in a period of two months on a daily basis. After cleaning raw data, the collection of features is poor as only 43 samples are available. This is, however, not uncommon in digitalization of clinical practice. The following rules are inferred by LLM and pass the FET test. The covering and error of a rule are denoted by *C* and *r*, respectively. The baseline data reported is not calculated through the GAN approach. The data are retrieved from a single patient monitored twice a day through all sensors available in a period of 3 months. The patient is a 45-year-old male. The quantity of information retrieved by IoMT is “longitudinal” as it is related to time-series physiological signals. Measurements propagate over time in such a way that every subject may constitute an independent stochastic process on its own, independently of the other subjects. It is also multivariate as it covers several sensors (as the ones coming from the outlined COPD pilot). This constitutes a novelty with respect to traditional augmentation studies, such as on electrocardiogram (ECG) [40], cough [41] or epileptic seizure [42] univariate traces. The augmentation of a set of IoMT subjects should be performed subject-by-subject [40,43] or between couples of signals [44]. The multivariate nature of an IoMT dataset, coming from a single subject, would preclude the joint augmentation of two subjects. This allows preserving the original correlation among the variables in each subject. In other words, the one-subject-many-signals setting introduces an additional order of dimensionality in data augmentation. IoMT drives a new information framework in ICT for medicine as clinical trials are typically based on a “vertical” basis, namely, clinical samples (e.g., exams at the hospital) do not cover a time longitudinal comparable with the one of IoMT (To some extent, this recalls the concept of precision medicine, in which the treatment should be specialized for the specific state of the subject under investigation (i.e., according to its genetic baggage); in that case, the joint mapping of clinical and genetic data drives the medical intervention, independently of the approaches chosen for the other subjects.).

**if**((heartrate<74)∧(diastolicpressure>67))**then** high (C = 43%) (r = 4.5%)

**if**((FEV1<2.23))**then** low (C = 41%) (r = 4.7%)

The resulting rules give intuitive indications about the quality of the breathing performance (through the PEF classification). One may argue the triviality of the knowledge extraction, as having good or bad breathing performance independent of body pressure and heart rate may be expected, even by non-expert in the field. However, the following statements should be highlighted. First of all, the synthesis made on all the features is useful in front of several sources of information (as in [5]); in this case, the LLM automatically concentrates the attention on a quite small subset of features. This is captured by both feature ranking and value ranking, not shown here, which give a clear indication of the most significant feature and ranges of values for each feature in mapping the output classes. This results in quite simple and clear rules for PEF modeling. Despite the fact that some rules may be intuitive for the expert of the field, thus posing doubts on the novelty of the knowledge extraction, some others may shed new light on the phenomenon in terms of variables involved and inherent thresholds.

## 5. Data Augmentation through GANs

In order to build synthetic data, to be coherent with the same (unknown) multi-dimensional probability distribution generating the available (real) dataset, the following methodology has been applied. In deep learning, the term autoencoder defines a neural network (NN) trained to approximately copy the input to the output. The model is usually forced to give priority to some specific aspects of the data. It is composed of two parts:

- An encoder function, h=f(x): it describes the code to represent the input,
- A decoder function, r=g(h), which produces the reconstruction.

Traditionally, they were used for dimensionality reduction and feature ranking. More recently, they have been applied to generative models. They may be thought of as a special case of feedforward networks, and they can be trained using the very same strategies. Learning a not-complete representation is commonly known as an under-complete autoencoder. It forces the autoencoder to capture the most salient features. On the other hand, giving too much capacity to the model leads to failing to learn anything useful (just a simple copy).

In this context, Generative Adversarial Networks (GANs) have the purpose of generating new, synthetic data (data augmentation). They learn the distribution of the training set and can generate new data never seen before. They generate the whole output all at once, differently from other types of generative models, such as recurrent NNs that generate one element at a time.

The underlying algorithmic principle is based on game theory. The game is posed between a generator network and a discriminator network. The generator uses the encoder–decoder scheme to build synthetic data. The discriminator infers the separation between real and synthetic data. More specifically, the discriminator learns to become better at distinguishing real from synthetic data; the generator learns to generate better data to fool the discriminator. In particular, we implemented a conditional GAN. We imposed a condition on both the generator and discriminator inputs. The condition is in the form of a one-hot vector version of the classes:

- In the generator, we associate the noise to a particular class;
- In the discriminator, we classify the rows as real or fake based on real and fake data and their corresponding labels.

**Figure 2:**
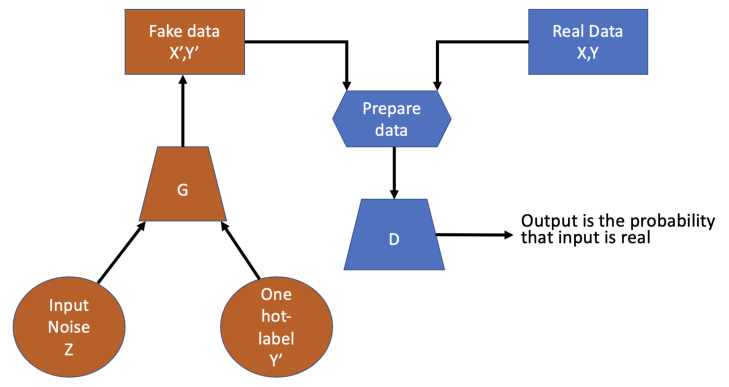
Conditional GAN schema.

Training the discriminator and generator in conditional GAN is similar to training a discriminator and generator in simple GAN. The only difference is that both the generated fake and real are conditioned with their corresponding one-hot labels. Figure 2 reports a schema about the implemented conditional GAN.

The optimization scheme of the training is formulated in order to achieve, at convergence, a game equilibrium in which the generator’s samples are indistinguishable from real data. A pseudo code of the GAN implemented is reported in Algorithm 1.
**Algorithm 1:** GANs pseudo-algorithm
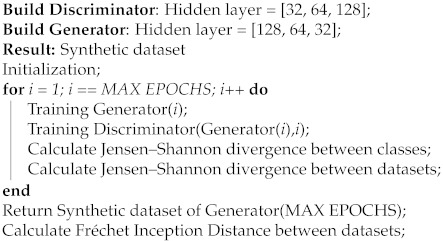


This has been typically applied in image processing, in which the applications are wide and innovative; an overview of the topic is discussed and presented in [45]. On the other hand, the application of GANs in the biomedical sector constitutes an open area of research [46].

### 5.1. Evaluation Metrics for the Proposed GAN

In general, deep learning neural network models are trained with a loss function until convergence. GANs lack an objective function, which makes it difficult to compare the performance of different models. Several techniques assess the performance of a GAN model based on the quality and diversity of the generated synthetic dataset. In this work, Jensen–Shannon (JS) divergence and the Fréchet Inception Distance (FID) score are used to validate the quality of the synthetic dataset based on recent research work [16,18]. Although the proposed works are focused on data augmentation of images, we have decided to apply some metrics and considerations as a starting point for our work.

The Jensen–Shannon divergence, given two probabilities distribution *P* and *Q*, is defined as:

(1) $$\sqrt{\frac{D_{KL}\left(P||m\right)+D_{KL}\left(Q||m\right)}{2}}$$

where *m* is the pointwise mean of *P* and *Q*.

In general, the FID score is applied to compare real images with fake generated images. The FID assumes both distribution *P* and *Q* are Gaussian with means $\mu_{P}$, $\mu_{Q}$ and covariance matrices $\delta_{P}$, $\delta_{Q}$, respectively. Then, the FID is defined as:

(2) $$FID\left(P,Q\right)=\left|\right|\mu_{P}-\mu_{Q}{\left|\right|}^{2}+T_{r}\left(\delta_{P}+\delta_{Q}+2\sqrt{\delta_{P}*\delta_{Q}}\right)$$

Lower FID values mean better quality and diversity of the data generated. FID is sensitive to mode collapse, so the distance increases with simulated missing modes. If the GAN model only generates one sample per class, the distance will be high.

### 5.2. Remarks

It is worth noting we are considering a bioinformatics context under an XAI paradigm. That means the nature of the information is different from images, and the aim of prediction is different as well: modeling the severity level of a disease (respiratory limitations, in this case). Moreover, a coordination between the synthetic dataset and XAI reliability is searched for. These points denote the challenge of the proposed work, as the largest majority of GAN applications are devoted to image analytics applications. Some preliminary results are proposed in this direction.

## 6. JS vs. FID vs. XAI

Once the data augmentation architecture and the evaluation metrics have been defined, the generation and evaluation of synthetic dataset through JS, FID and XAI performance is elaborated. As already said, the process is considered successful by the GAN if the generator fools the discriminator, namely, the generated data are indistinguishable from the real data. Together with JS and FID, which give an indication of the quality of the synthetic dataset, an XAI validation process is introduced. It consists of validating the significance of the rules generated on the corresponding synthetic dataset. The possible approach to achieve this goal is to firstly rely on the statistical validation of the rules (obtained by the model derived on the synthetic dataset). Then, the filtered rules are tested on the real dataset.

Some preliminary definitions are needed to avoid confusion between the models generated on the real or on the synthetic dataset. Let the baseline and candidate augmented models name the sets of rules derived and be statistically validated on the original (real) dataset and on the generated one, respectively. The candidate augmented model is further tested on the original data, thus giving rise to an additional model, the augmented model, whose statistical validation acknowledges if the final test is passed. That means the candidate augmented model becomes valid only if the corresponding augmented model returns statistically valid rules on real data.

Therefore, the number of rules validated by the Fischer test, accuracy and F1 score are quality metrics available at the end of the analysis, namely, after having trained XAI on the synthetic dataset, validated it through the Fisher test and tested it on the real one. The aim is to discover potential alignments among JS, FID and XAI. More specifically, predicting XAI quality through JS or FID would be very relevant as it may give an indication of the quality of the XAI model without testing it in practice.

## 7. Obtained Results

**Figure 3:**
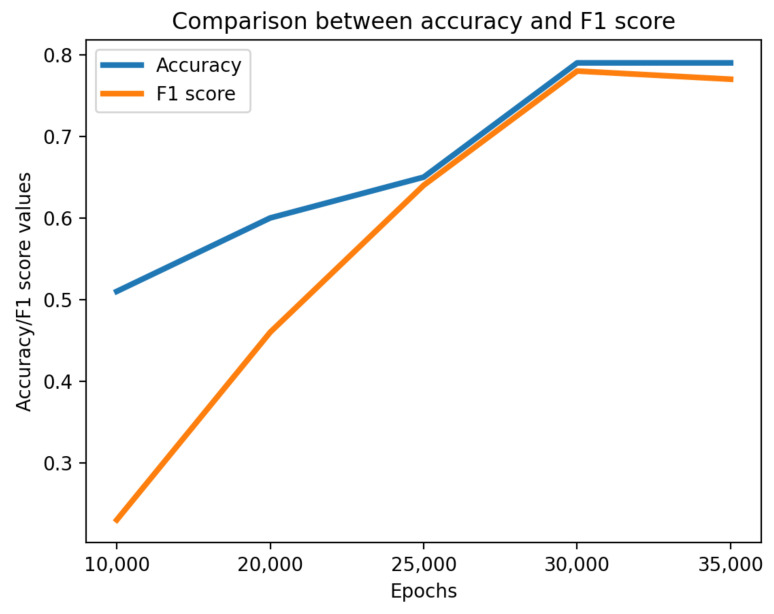
Comparison between accuracy and F1 score.

Several independent runs of data augmentation are provided with different numbers of epochs (the rest of the neural structures are left untouched). One thousand synthetic samples were generated. The inherent results are presented in Table 1 and Figure 3.

Unfortunately, JS does not provide stable indications on the quality of XAI, but the FID does. The optimal setting in terms of minimum FID, highest number of validated rules, best accuracy and F1 score is achieved with 3000 epochs. That means FID may anticipate the quality of XAI, thus avoiding the need for its continuous testing over all the candidate data augmentation runs.

The generated rules in correspondence of the optimal setting, whose significance is in line with the baseline (condition on FEV1), are reported below. The introduction of the MAP feature, not present in the baseline, may shed new light on the knowledge extraction process at hand. More specifically, the baseline and the augmented model may be posed to the attention of a clinician for further validation. This subsequent validation step is one of the most important issues we have in mind for the near future.

**if**((FEV1>2.18)∧(MAP>90.39))**then** high (C = 27%) (error = 3.58%)

**if**((FEV1<=2.23)∧(MAP<=104.18))**then** low (C = 41%) (error = 4.93%)

The machine learning model develops rules itself. Those rules are further statistically validated and then later posed to the attention of clinicians. The entire chain of analysis is made by AI without human intervention. The task of writing several candidate rules may be tedious, but that is where AI comes into play as it provides a tool for automatic rule generation.

## 8. Conclusions

In this paper, we investigated the adoption of generative adversarial networks (GANs) and an explainable AI algorithm on data retrieved by IoMT devices.

Moreover, the evaluation of the synthetic dataset generated with the GAN is performed by using the LLM algorithm compared with the real information. The results obtained show how the synthetic dataset is aligned with the real dataset, further demonstrated by the rules obtained through the LLM algorithm.

Further work on the topic may also be directed to the adoption of other IoMT sensors to monitor health parameters of the patient. Moreover, the combination of environment and health sensors will be evaluated in order to demonstrate if the environment affects disease. Another future work is to extend this approach by involving more patients in order to increase the precision of the algorithm and the generalization of the synthetic data. Similarly, adversarial machine learning techniques could be discussed for the dataset and algorithms in order to evaluate and validate the robustness of the platform. Finally, due to the many rules generated by machine learning algorithms, we will investigate other statistical validation approaches or automatic algorithms to assist human decision (e.g., rule distance). 

## Figures and Tables

**Table 1:** Obtained metric results for data augmentation.

Epochs	JS Real-Fake Data	JS between Classes	FID	Rules Accepted by FET	Accuracy	F1 Score
10,000	0.42	0.52	320.78	0	0.51	0.23
20,000	0.67	0.28	97.97	1	0.60	0.46
25,000	0.59	0.44	21.05	2	0.65	0.64
30,000	0.62	0.42	11.87	2	0.79	0.78
35,000	0.59	0.60	88.32	1	0.79	0.77

## Data Availability

The data presented in this study are openly available in a Kaggle repository: https://www.kaggle.com/cnrieiit/bpco-dataset-based-gans-for-iomt (accessed on 26 May 2021).
